# Effects on cognition of regional astrogliosis assessed by ^18^F-SMBT-1 PET in non- demented individuals

**DOI:** 10.21203/rs.3.rs-10449518/v1

**Published:** 2026-07-31

**Authors:** Laura-Alejandra Ramirez-Tirado, Ann D Cohen, Brian J Lopresti, Milos D Ikonomovic, Alexandra Gogola, Cristy Matan, Xuemei Zeng, Thomas K Karikari, Oscar L Lopez, Beth E. Snitz, C Elizabeth Shaaban, Victor L Villemagne

**Affiliations:** University of Pittsburgh

## Abstract

**Background:**

Increased peripheral inflammation and astrogliosis, as assessed by glial fibrillary acidic protein (GFAP) associates with worse cognition. GFAP, however, does not provide brain spatial information. ^18^F-SMBT-1, a tracer that binds to overexpressed MAO-B in reactive astrogliosis, may help overcome this limitation. Neuroprotective growth factors (e.g. BDNF, VGF, VEGFA, VEGFD, IGF1R and IGFBP7) usually accompany astrogliosis and could mediate the pathways between AD pathology and cognitive impairment. We evaluated the relationships among peripheral inflammatory markers, growth factors, astrogliosis, and cognitive function and explored whether the relationship of astrogliosis with cognition could be mediated by these growth factors.

**Methods:**

In a sample of older adults without dementia, we evaluated the relationship of astrogliosis with overall and domain-specific cognitive performance (verbal memory, visual memory, executive function and attention). ^11^C-PiB and ^18^F-SMBT-1 radiotracers were used to assess Aβ status and astrogliosis, respectively. The NULISAseq CNS disease panel was used to assess GFAP, markers of peripheral inflammation and selected growth factors. We used multivariable robust linear regression models to test the relationship of peripheral inflammation and astrogliosis and cognition. Later, we ran causal mediation analyses stratified by Aβ status to address the effects of BDNF and IGFR1 on the relationship between astrogliosis and cognition.

**Results:**

Ninety-four non-demented participants were included (mean age: 68 ± 7 yrs, 52% women, 89%, White); among them, 76 were classified as A− and 18 as A+. ^18^F-SMBT-1 binding in precuneus was higher among A+ individuals. Also, among A+ individuals, regional ^18^F-SMBT-1 binding was associated with worse verbal and visual memory and executive function. Lastly, we found that IGFR1 but not BDNF significantly mediated (suppressed) the association of ^18^F-SMBT-1 with worse visual memory, but only among A+ individuals (p<.0001).

**Conclusion:**

In this non-demented population of older adults with low levels of Aβ pathology and neuroinflammation, astrogliosis related with worse cognitive functions, but only among A+ individuals. Further, IGFR1 may play a suppressive role decreasing by more than 50% the effect of astrogliosis on worse visual memory.

## Introduction

Alzheimer’s Disease (AD), the leading cause of dementia, is characterized by the accumulation of the pathological proteins Aβ and hyperphosphorylated tau [1]. However, these two proteins do not fully explain the variation we see in the range of clinical expression of the disease. The need to better understand clinical heterogeneity in AD has motivated our group and others to evaluate other factors such as neuroinflammation [2][3], [4], [5].

Neuroinflammation, characterized mainly by the activation of microglia and astrocytes, along with the release of cytokines and growth factors, contributes to AD pathology [6], [7]. Astrogliosis, the dynamic process by which astrocytes respond to central nervous system damage, is characterized by astrocytic activation, expression of glial fibrillary acidic protein (GFAP), and overexpression of monoamine oxidase-B (MAO-B) [8], [9]. Astrogliosis, as assessed by plasma GFAP, has been associated with worse overall cognition, and a few studies have also shown inverse associations of GFAP with domain-specific cognitive performance [10], [11]. While previous studies have provided support for the role of astrogliosis in cognitive decline, a more mechanistic approach is needed to guide effective therapeutic interventions.

In vivo neuroimaging with PET tracers targeting MAO-B could help advance the regional understanding of astrogliosis in AD. MAO-B is an enzyme expressed in the outer mitochondrial membrane that catalyzes the oxidation of several monoamine neurotransmitters [12]. It is also expressed on catecholaminergic neurons that are critical for various aspects of cognition including memory and learning, executive function and attention. However, the use of extant MAO-B tracers such as ^11^C-DED is constrained by its irreversible kinetics, low sensitivity and short half-life time. Nonetheless, a new MAO-B tracer,^18^F-SMBT-1, has been developed to assess astrogliosis in vivo [12], [13]. This radiotracer has shown reversible kinetics and robust binding across brain regions known for early Aβ deposition [14]. Furthermore, ^18^F-SMBT-1 binding has been shown to have good correlation to GFAP when all clinical groups across the AD spectrum are assessed together [15].

Astrogliosis, however, does not occur in a vacuum. The cellular neuroinflammatory response is accompanied by the release of peripheral inflammatory factors, cytokines and growth factors that could mediate important transduction pathways such as TrkB pathway, which is relevant for the neurotrophin signaling cascade regulating neuronal survival, growth and synaptic plasticity and could partially explain the role of astrogliosis on future cognitive decline[16], [17]. Furthermore, the study of some growth factors, whose functions are regulated through the TrkB receptor or related downstream cascades (such as PIP/Akt, MAPK/ERK and PLCγ) could also provide a more complete picture of the different mechanisms that interact with astrogliosis, such as angiogenesis, synaptic remodeling, and metabolic regulation. In fact, some growth factors such as brain-derived neurotrophic factor (BDNF), insulin-like growth factor receptor 1 (IGFR1) and vascular endothelial growth factors (VEGF) exert their functions through TrkB receptors or related downstream cascades and have been positively associated with brain volume, hippocampal perfusion, cerebral blood flow, and/or synaptic connections [18], [19], [20], [21], [22], [23].

Thus, the aim of this study was to assess the relationship between regional astrogliosis assessed via ^18^F-SMBT-1 PET and selected growth and inflammatory factors (BDNF, VGF, VEGFA, VEGFD, IGF1R,IGFBP7, CRP, IL-6, TNF, IL-4, IL-10 and IL-13) with overall cognition and domain-specific cognitive performance (verbal and visual memory, attention, and executive function) among non-demented participants. Given the importance of Aβ status, we also evaluated if the associations of astrogliosis with cognition were moderated by this third variable. We hypothesized that astrogliosis would be associated with worse overall cognition and worse domain-specific cognitive performance, particularly among Aβ + individuals. In addition, we hypothesized that growth factors would be associated with better overall and domain-specific cognitive performance across groups. Lastly, in a post hoc analysis we assessed if growth factors mediated the association between astrogliosis and cognition.

## Methods

### Study population

We used data from an ongoing program project grant (PPG) entitled The role of astrogliosis in aging and the pathological and clinical progression of Alzheimer’s disease (INROAADS). The project is a longitudinal observational study that aims to contribute to understanding of pathophysiological mechanisms that lead individuals to develop dementia, especially AD. Briefly, the current study involves a cross-sectional analysis of the first 94 participants without dementia enrolled in this ongoing project. All participants signed informed consent and underwent baseline assessments involving: 1) review of their medical record, 2) general interviews, 3) clinical physical examination and neurological evaluation, 4) an extensive neuropsychological battery 5) blood sampling and 6) a neuroimaging protocol.Participants were excluded if they were taking relevant medications such as MAO-B inhibitors, monoclonal antibodies, immunosupressant treatment, steroids, or had taken chemotherapy within one year. Likewise, participants were excluded if they had relevant hematological, heart, kidney or liver disease as well as if they had schizophrenia, bipolar disorder or any other relevant CNS disease. Finally, smokers who quit smoking within the last three months or individuals with alcohol or drug dependence were also excluded.

#### Neuropsychological examination

Participants were assessed for their overall cognition using the Montreal Cognitive Assessment (MoCA) test, a brief 30 point neurocognitive test[24]. Word list delayed recall test was used to evaluate verbal memory[25]. In this test, the person is asked to spontaneously recall as many words from an original list of 10 words after a distractor task of 5–10 minutes. The Benson complex figure (BCF) delayed recall was used to assess visual memory [26]. In this part of the test, the participant is asked to draw the figure that was first copied from the example from memory after a 10–15 min interval. We assessed attention using digit span forward[27], a simple test where the participant is asked to repeat increasing length sequences of numbers in the same order. Lastly, we assessed executive function using letter fluency (with the letters F, A, and S)[28] and the clock drawing test[29], in which participant is asked to draw a clock face, placing all the numbers on the clock and setting the time at 10 after 11.

#### Cognitive diagnosis

The diagnosis of mild cognitive impairment (MCI) or normal cognitive function was based on baseline assessments and adjudicated by a consensus process following the ADRC/National Alzheimer’s Coordinating Center (NACC) MCI guidelines for MCI classification (Dubois et al., 2016). Participants' cognitive status was classified by evaluating their cognition considering age and education. A participant was diagnosed with MCI if: 1) the participant did not have normal cognition for age based on reviews of all clinical and cognitive information; 2) the participant did not meet the criteria for dementia; 3) there was evidence of cognitive decline at the time of the assessment; and 4) the participant otherwise showed normal functional activities. Normal cognition was defined by exclusion, considering that the participant did not meet the criteria for MCI or dementia, and that the participant had normal cognition for their age and education, based on a review of all clinical and cognitive information.

#### Multimodal neuroimaging protocol

##### MRI.

Sagittal T1-weighted magnetization-prepared rapid acquisition with gradient echo (MPRAGE) and T2-weighted fluid-attenuated inversion recovery (FLAIR) MR images were acquired using a 3.0T Siemens Prisma scanner (Siemens Healthcare). MPRAGE MR images were processed with FreeSurfer (FS) v7.1.1 to generate a brain parcellation atlas for sampling the PET images[30], [31]

##### PET.

All PET images were collected on a Biograph mCT (TrueV) (Siemens Healthcare) and reconstructed as described elsewhere[30]. All PET images were motion-corrected as needed based on visual determination and registered to their corresponding MR images.

Aβ PET imaging was performed using ^11^C-Pittsburgh Compound B (PiB), which was produced under an approved United States Food and Drug Administration (FDA) Investigational New Drug (IND) application (IND#72200). PiB PET images were collected and summed 50–70 minutes post-injection[32].

SMBT-1 (IND #166977) was produced as previously described, under an approved FDA IND application (IND#166977).[33] SMBT-1 PET images were collected and summed 70–90 minutes post-injection[33].

FS-identified cerebellar gray matter was used as the reference region to convert the PiB and SMBT-1 images from standard uptake values to standardized uptake values ratios (SUVRs)[32], [33]. PiB SUVR images were transformed into PiB Centiloid (CL) images using a site-specific validated transformation[34].

PET outcome images were sampled using the FS brain parcellation atlases derived from the corresponding MRIs. Radiotracer-specific composite regions of interest were combined via volume-weighting for each PET image. PiB Centiloid images were sampled with a global region, comprised of anterior cingulate, anterior ventral striatum, superior frontal, orbitofrontal, insula, lateral temporal (inferior and middle temporal), parietal, posterior cingulate, precuneus[32]. SMBT-1 SUVR images were sampled in regions of early Aβ accumulation like the posterior cingulate.

#### Growth factors and markers of peripheral inflammation

Plasma samples included in this study (n = 94) were collected in 2025 using lavender top K2EDTA tubes. The tubes were centrifuged 2000 x g for 10 minutes. Plasma was then aliquoted into cryovials for long-term storage at − 80°C. Samples were analyzed at the University of Pittsburgh using the NULISAseq CNS kit and were processed according to manufacturer instructions. The peripheral markers of inflammation that we studied were IL-6, CRP, TNF, IL-4, IL-10 and IL-13 along with GFAP. The growth factors assessed were BDNF, VGF, VEGFA, VEGFD, IGF1R and IGFBP7.

### Confounding variables

Age, sex and years of education were self-reported. These confounding factors were chosen based on previous studies evaluating astrogliosis with ^18^F-SMBT-1 in AD[15].

### Statistical analysis

We performed descriptive analysis to compare characteristics between non-demented A− participants vs. non-demented A+ participants. We also describe the differences in ^18^F-SMBT-1 binding across 22 regions and on cognitive tests stratified by PET Aβ status. Continuous variables were summarized as means with standard deviations and visualized with box plots. Meanwhile, categorical variables were tabulated as frequencies with percentages. To test differences across groups, we used t-tests for continuous variables and Chi-square tests for categorical variables.

To assess the relationships of astrogliosis (via ^18^F-SMBT-1), of peripheral inflammation and of growth factors with overall cognition and domain-specific cognitive tests, we used robust linear regression models adjusted for age, sex, and years of education. In addition, we ran a secondary analysis of how GFAP associated with cognition to confirm what we found with ^18^F-SMBT1. All analyses were stratified by Aβ status. Results of fully adjusted regression models are presented as coefficients with 95% CI.

Finally, after we ran the main analyses on how astrogliosis (via ^18^-FSMBT-1) associated with cognition, we chose to ran a post hoc analysis to test the mediation effect of selected growth factors with visual memory using a pragmatic approach. Instead of addressing all the cognitive domains, we chose visual memory for the post-hoc analyses, because among the different domains explored, visual memory was the most consistently affected by astrogliosis (via ^18^F-SMBT-1). Further, we chose to evaluate BDNF and IGFR1 as mediators, because among the six growth factors explored, these are the once with a more biological plausibility to be involved according to current literature and among the the six growth factors evaluated, both showed at least the same direction of effect in A− and A+ participants in relation to cognition. To that aim we ran separate causal mediation analyses for BDNF and for IGF stratified by PET Aβ status (A−/A+). In the mediation analysis, we estimated the total effect (TE) of the association between astrogliosis and cognitive function, which comprises the sum of the natural direct effect (NDE) of astrogliosis on cognitive function without taking into account a mediator and the natural indirect effect (NIE) of astrogliosis on cognitive function taking into account each of the growth factor as mediators.

We accounted for multiple comparisons by controlling the false discovery rate using the Benjamini Hochberg procedure for statistically significant associations. Analyses were done using Stata V.18. and SAS 9.4, and plots were done with Stata V.18 and R 2025.05.1 + 513.

## Results

A total of 94 participants were included in the analytic sample. Their mean age was 65 years, 48% were male, most of them were non-Hispanic white (89%), and they were highly educated (mean years of education: 16). The prevalence of former smokers was 36%, diabetes 14%, hypertension 51%, hypercholesterolemia 60%, and the mean MoCA score was 26.2 (Table 1).

A total of 76 participants were A−, and a total of 18 participants were A+ individuals. We did not find differences in clinical and sociodemographic characteristics by A status. (Table 1). A+ individuals presented with higher ^18^F-SMBT-1 binding in precuneus compared to A− participants. We did not find any other significant differences in ^18^F-SMBT-1 binding across the various brain regions studied when contrasting both groups (**Supplementary Fig. 1**).Regarding cognitive test scores, we found that compared to A− individuals, A+ individuals had a lower score in word list delayed recall test (p=.015. We found no other significant differences by A status (**Supplementary Fig. 2**).

### Astrogliosis and cognitive performance

Primary analyses: ^18^F-SMBT-1. ^18^F-SMBT-1 binding across the different brain regions was not associated with overall cognition as assessed by MoCA in A− nor A+ participants (Fig. 1). However, we found that among A+ individuals, higher ^18^F-SMBT-1 binding across brain regions such as inferior parietal, superior parietal, superior temporal, supramarginal gyrus, and DLPFC was associated with worse executive function as assessed by letter fluency. Likewise, we found that among A+ individuals, higher ^18^F-SMBT-1 binding across most of the brain regions including anterior cingulate, entorhinal cortex, inferior parietal, inferior temporal, lateral occipital, cuneus, middle temporal, posterior cingulate, precuneus, superior parietal, superior temporal, supramarginal gyrus, DLPFC, VLPFC and orbitofrontal cortex was associated with poorer visual memory as assessed by Benson complex figure test with effect sizes ranging from – 1.36 to −2.92. This finding related to memory was further corroborated when we assessed word recall delay. As such, in most brain regions higher ^18^F-SMBT-1 binding associated with poorer verbal memory as measured by delayed recall of the word list; effect sizes ranged from – 1.39 to 5.75. In addition, higher ^18^F-SMBT-1 binding among A+ individuals was also associated with worse executive function as assessed by F.A.S but not by clock drawing test in several brain regions. Associations ranged from – 1.78 to −1.52. Lastly, ^18^F-SMBT-1 binding showed a trend towards worse attention evaluated by digit span forward (but associations were not statistically significant) (Figs. 1A & 1B **and Supl.** Table 1).

Secondary analyses: GFAP. Plasma GFAP was only associated with worse executive function (via clock drawing test) in A+ (−1.34; p=.034) (Figs. 1A & 1B **and Supl.** Table 1).

#### Growth factors and peripheral inflammatory markers and cognitive performance.

**Figure 2.**
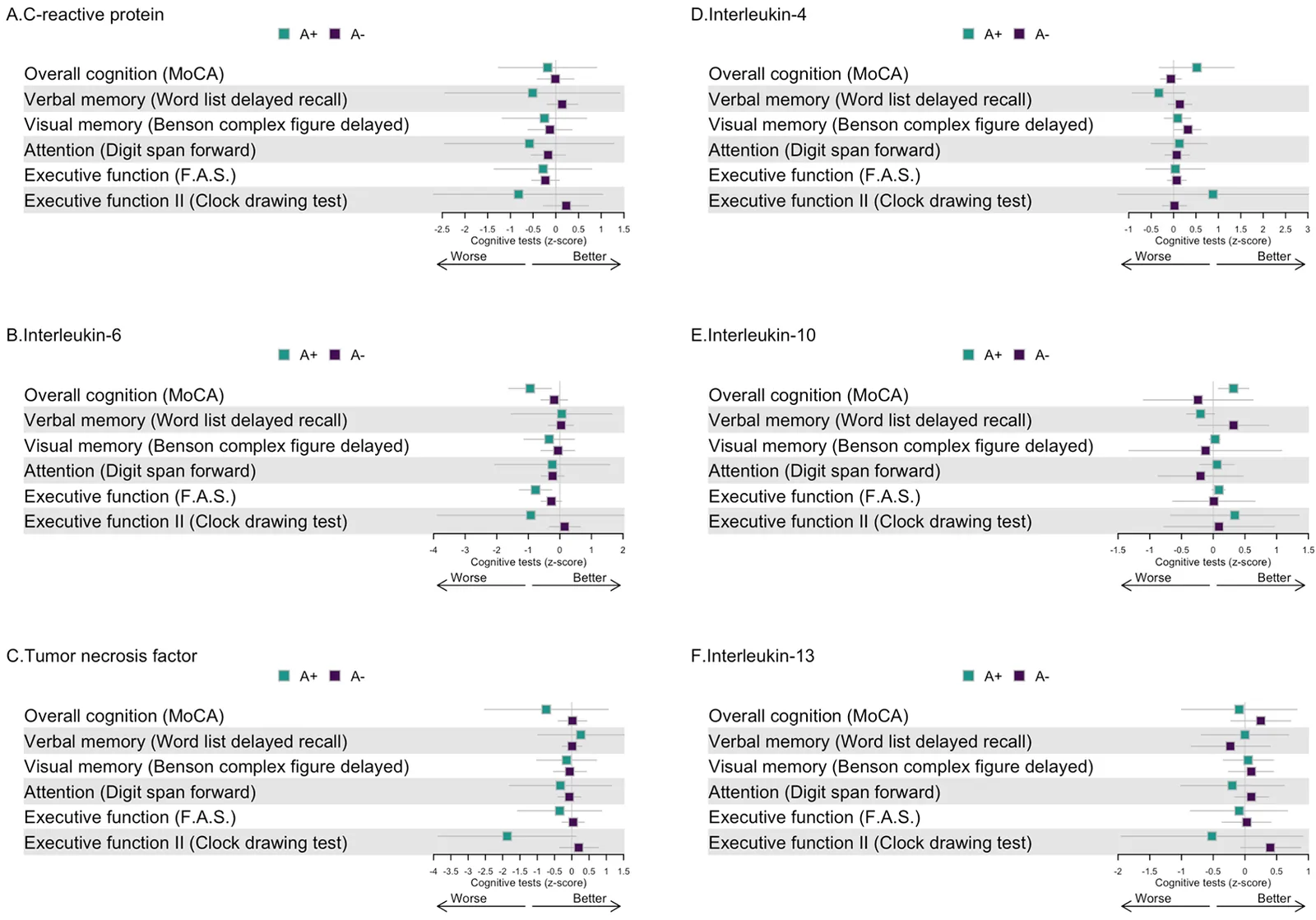
Relationship of pro- and anti-inflammatory cytokines and cognitive functions among non-demented A− and non-demented A+ participants.

**Figure 3.**
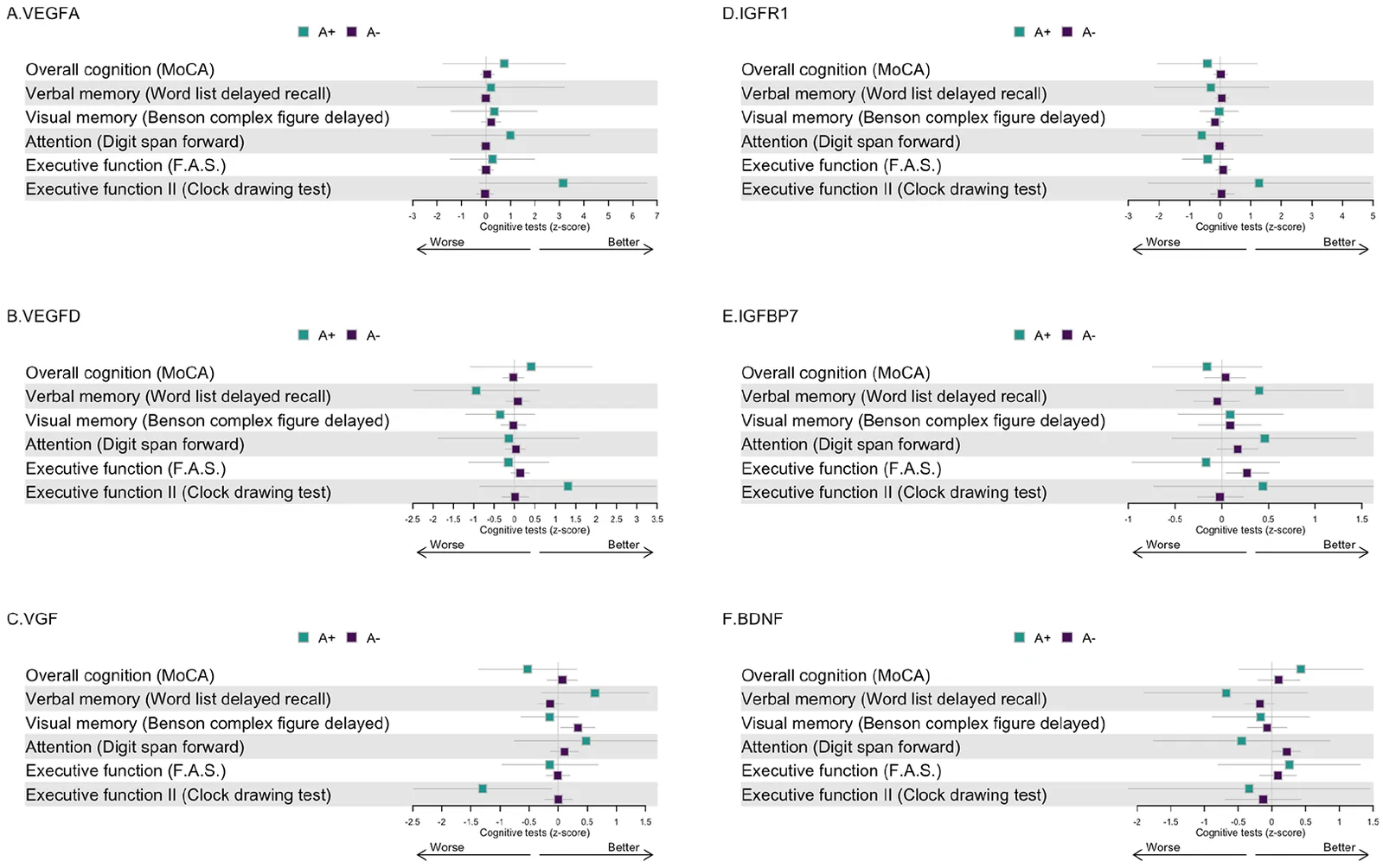
Relationship of selected growth factors and cognitive functions among non-demented A− and non-demented A+ participants. Abbreviations: VEGFA:Vascular endothelial growth factor A;VEGFD:Vascular endothelial growth factor D; VGF: Nerve growth factor; IGFR1:Insulin growth factor receptor 1, IGFBP7:Insulin groth factor binding protein 7; BDNF:Brain derived neurotrophic factor

We found that greater IL-6 was significantly associated with worse overall cognition and executive function in A+ participants. As such each standard deviation greater IL-6 level was associated with 0.94 SD lower MoCA score (p = 0.018) and 0.77 SD lower F.A.S. score (p = 0.014). No other associations were significant. By contrast, also among A+ individuals, IL-10 associated with better overall cognition, and executive function. Each SD greater IL-10 level was associated with a 0.32 SD greater MoCA score (p = 0.019), and a 0.09 SD greater with a trend of better FAS score (p = 0.078). No other associations were significant. Similarly, but only among A− participants, greater IL-4 associated with better visual memory. As such each standard deviation greater IL-4 was associated with 0.32 SD greater Benson complex figure delay score (p = 0.034).Regarding growth factors, in A− individuals VGF associated with better visual memory as assessed by BCF. As such, each SD greater in VGF level was associated with 0.34 SD greater, respectively in BCF score (p = 0.28). Also, among A− greater IGFBP7 was associated with better executive function. Each SD greater IGFBP7 level was associated with a 0.27 SD greater F.A.S. score, p = 0.017. No other associations were significant in A− or A+ perticipants (Figs. 2 and 3 **and Suppl. Table 2**).

### Growth factors as mediators of the relationship between astrogliosis and cognition

When we explored whether neuroprotective growth factors could mediate the effects of astrogliosis on cognitive impairment, we found that BDNF did not mediate the association of astrogliosis either assessed through ^18^F-SMBT-1 (NIE p = 0.83) or GFAP (NIE p = 0.73) with worse visual memory among A+ individuals (**Supl.** Figure 1).

**Figure 4.**
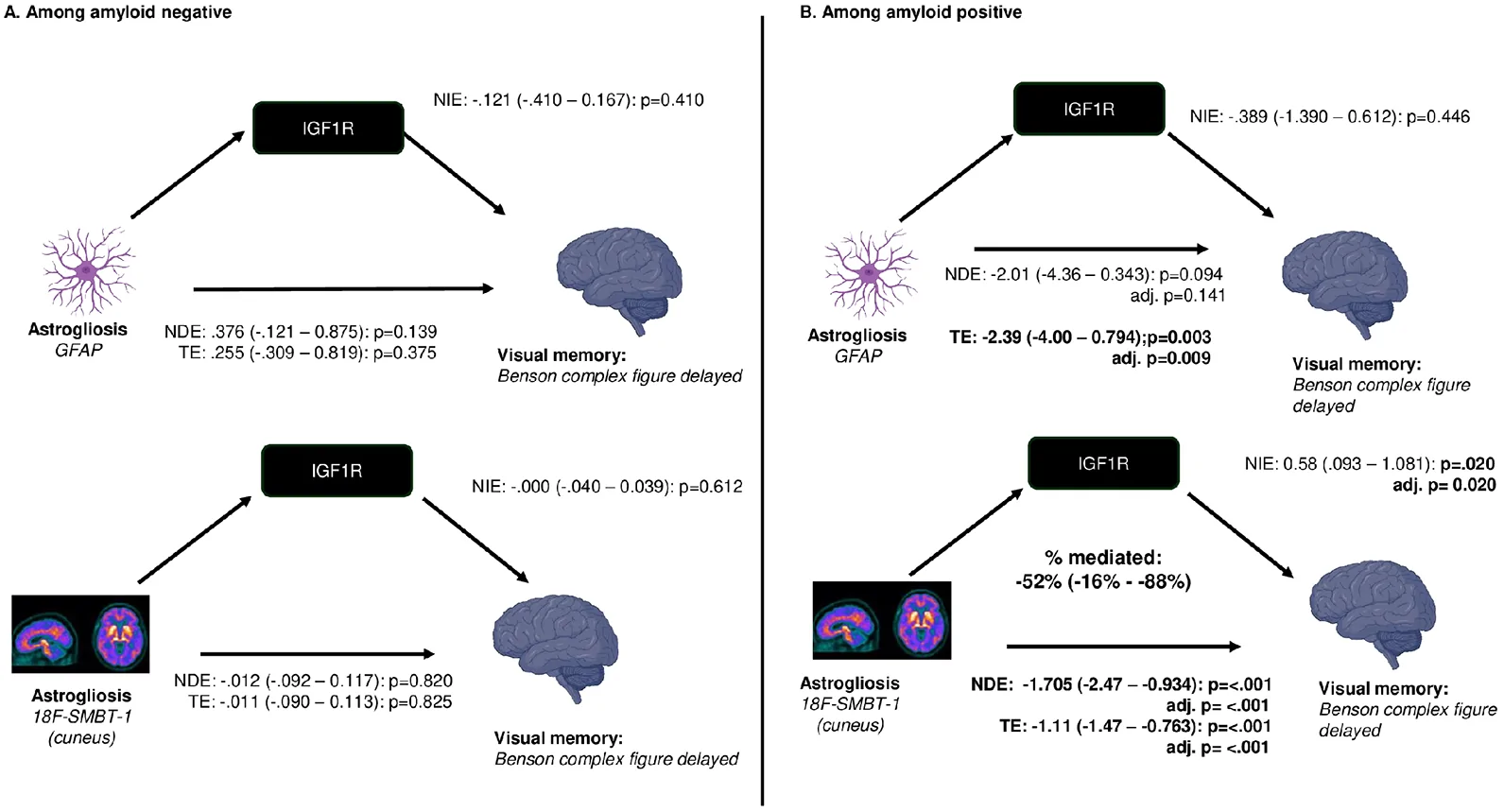
Mediation analysis of IGFR1 on the relationship of astrogliosis to memory and executive function. **Notes:** * Adjusting for age, sex, education. **Abbreviations:** NIE: natural indirect effect; NDE: Natural direct effect, TE: total effect: GFAP: Glial fibrillary acidic protein. **Notes:** * Adjusting for age and education. **Abbreviations:** NIE: natural indirect effect; NDE: Natural direct effect, TE: total effect: GFAP: Glial fibrillary acidic protein. Adjusted p-values for multiple comparisons w/ Benjamini-Hochberg method.

By contrast, when we explored the mediation effect of IGFR1 on the relationship of astrogliosis with visual memory we found that IGFR1 showed a suppressive mediation effect on the association of ^18^F-SMBT-1 astrogliosis with worse visual memory (NIE p = 0.02; FDR adjusted p = 0.02) (Fig. 4). However, IGFR1 did not show a suppresive mediation effect in the relationship of GFAP with worse visual memory (NIE p = 0.45).

## Discussion

In this baseline cross-sectional assessment of nearly one-hundred participants from INROAADS, with a low prevalence of AD pathology (~ 20%) we found that astrogliosis as assesed by increased ^18^F-SMBT-1 PET was greater in precuneus and was associated with worse perfromance in verbal and visual memory and executive function which have been consistently described as the first cognitive domains affected at the early phases of the disease[35], [36]. These results are consistent with previous study that found that increased ^18^F-SMBT-1 binding associate with worse cognition and that increased ^18^F-SMBT-1 binding explains around 25 to 30% of the variance in cognitive tests such as MMSE [37]. However, unlike previous study, we did not found that ^18^F-SMBT-1 binding associated with worse overall cognition as assessed by MoCA. In other words, astrogliosis seems to be more relevant to specific cognitive domains at early phases of AD.

In line with this, greater ^18^F-SMBT-1 binding was associated with worse performance in visual memory and verbal memory and executive function. Further, among the different brain regions affected, we found that greater ^18^F-SMBT-1 binding in the bank of the superior temporal sulcus (BANKSSTS), in the inferior and superior parietal cortex and in DLPFC consistently associated with worse performance in these three domains. Our findings are consistent with previous reports in AD. The BANKSSTS, for instance, is one of the top-Aβ affected regions in AD[38], [39]. Astrogliosis in this cortical region has been reported to associated with worse cognitive trajectories[39]. Inferior temporal cortex is a crucial region in the brain for high-level object recognition, facial perception and visual memory [40]. Elevated GFAP in temporal lobe regions, as measured by ELISA in tissue homogenates, has been previously shown to be associated with worse memory performance regardless of Aβ and tau pathology [41]. Regarding DLPFC, this is a critical region for working memory, as it holds and manipulates short-term information. Further astrocytes in this region are relevant for maintaining proper neurotransmitter balance (like glutamate and GABA) and secreting growth factors as BDNF. Indeed, previous reports have shown that this region relies heavily on BDNF/TrkB signaling to maintain the persistent firing of neurons needed to hold information “in mind”[42], [43]. However, persistent astrogliosis can lead to dysregulation of BDNF and impaired memory consolidation.

In addition, other regions such as precuneus, anterior and posterior cingulate (PCC), and orbitofrontal cortex were consistently associated with worse verbal and visual memory but not with worse executive function. Further, precuneus also was the only brain region that has greater ^18^F-SMBT-1 binding among A+ participants as compared to A− individuals. Our results are in line with previous studies. Precuneus is a highly active hub for brain’s default mode network (DMN), that is critical for memory retrieval, internally-directed thought and episodic memory processing[44]. Precuneus and posterior cingulate cortex are both well-known areas associated with early Aβ accumulation [15], [45], especially in a scenario of low AD pathology [13]. Further, since these regions (precuneus and posterior cingulate) are one of the most early regions affected by Aβ, the lack of association of ^18^F-SMBT-1 binding in this regions with executive function may reflect an early contribution of astrogliosis in those regions in the evolution of AD. Astrogliosis in the PCC acts as a bridge between protein buildup (such as Aβ and tau tangles) and clinical memory loss. Previous PET studies have shown that elevated ^18^F-SMBT-1 binding in this region correlated with poorer performance in episodic memory tasks[46].

We also found that GFAP associated with worse executive function. However, contrary to previous studies we failed to showed an association of GFAP with overall cognition or with other cognitive domains. The discrepancy in the findings between GFAP and SMBT-1 may indicate that SMBT-1 and plasma GFAP are not necessarily representing the same type of astrogliosis, with regional SMBT-1 as a potential earlier indicator of reactive astrogliosis. Indeed, SMBT-1 reflect regions of overexpressed MAO-B in reactive astrocytes in the vicinity of dense-core Aβ plaques and support the rationale of using PET tracers. Further, overexpression of MAO-B by reactive astrocytes has important pathophysiological implications. As such, MAO-B overexpression by reactive astrocytes lead to an increase of reactive oxygen species and lipid peroxidation in the setting of dense-core Aβ plaques facilitating dystrophic neurites. On the other side, MAO-B overexpression could also promote GABA synthesis, which in turn leads to memory impairment[47]. Indeed, aberrant GABAergic inhibition has been shown as a pathway for memory impairment in AD mouse models[48].

In terms of peripheral markers of inflammation and growhth factors, we found somewhat consistent results. As such, we found that similar to previous studies, IL-6 associated with worse overall cognition[49], [50], [51], while other, poorly studied, yet relevant anti-inflammatory cytokines in AD such as IL-10 and IL-4 were associated with better cognitive outcomes[52], [53]. Although we do not have more elements to disscuss the potential pathological associations of IL-4 with cognition, it is important to remember that it could mediate the hippocampal-BDNF signaling pathway to impair reference memory[52].

In order to get a better understanding of the relationship between astrogliosis and worse visual memory, we ran a post hoc analysis testing potential mediating effects of neuroprotective growth factors such as BDNF and IGFR1 on this association. BDNF downregulation has been associated with greater AD pathology, while its upregulation is associated with amelioration of neuronal loss and dendritic atrophy mediated by Aβ pathology [54], [55]. Meanwhile, [39], [40] IGFR1 represent markers of glucose metabolism and have been associated with oxidative stress, neuroinflammation and neurogenesis [56], [57]. Both growth factors have been consistently associated with synaptogenesis and memory formation[54], [55], [58]. We hipotezised that astrogliosis, as an homeostatic response, may lead to the production of inhibitory mediators to decrease neuroinflammation in the brain. As such, our first potential mediator to exert this role was BDNF and in second term IGFR1.

Interestingly, contrary to what we may expect, we found that a suppressive mediator effect was only present among A+ individuals. Second, rather than BDNF, IGFR1 was the primary factor for this suppressive effect, accounting for more than half of the supression. Third, the IGFR1 suppressive mediation effect from astrogliosis to worse visual memory appears when astrogliosis is assessed by ^18^F-SMBT-1 but not with GFAP. The lack of mediating effects from GFAP coupled with the fact that the effects of astrogliosis on worse memory were only found among A+ individuals may reflect an active homeostatic response to Aβ plaques and a differential role of GFAP + and ^18^F-SMBT-1 + reactive astrocytes. Indeed, compared to the ubiquitous expression of reactive astrocytes expressing GFAP, reactive astrocytes surrounding Aβ plaques overexpress MAO-B. These reactive astrocytes mediate GABAergic inhibition due to the increased of GABA release and impairment of long-term potentiation (LTP) that in turn inhibit synaptic release and lead to memory impairment[48]. Indeed, clinical interventions aimed to tacked impaired GABAergic inhibition such as selegiline have shown to rescue LTP as well as memory and learning impairment, and although meta-analyses do not support its long-term use, they support its benefit over memory[59]. Therefore, ^18^F-SMBT-1 could be more specifically reflecting astrogliosis relevant to AD pathology [12]. In addition, the mediation of the effect by IGFR1 rather that BDNF suggests that, IGFR1 may be the real mediator, since in pathophysiological terms, IGFR1 is the main regulator of TrKB receptors, which constitute the main receptor by which BDNF exerts its neuroprotective function [57], [60]. Further, it seems possible also that the large mediated effect of IGFR1 could be to some extent due to IGF-1 signaling potentiating GABAergic inhibition[61]. Indeed, TrkB signaling has a direct role in controlling the number of GABAergic synapses[62]. In other words, the IGFR1 mediated pathway could reflect the neuroprotective role of IGFR1 in suppressing the effects of Aβ-related astrogliosis on memory function. If true, stimulation of IGFR1 could be potentially used to improve cognitive deficits. However, the results of IGFR1 should be taken with caution; since current evidence of IGFR1 and cognition is mixed, and some studies have even shown a deleterious effect of IGFR1 on cognition, since IGFR1 effects are multifaceted [63]. As such, although IGF1R declines with age and exercise induced increase of IGFR1 has been associated with better cognitive outcomes [63], [64], there is a need to further assess this association.

Some strengths of our study are worth mentioning. First, we are addressing the baseline data of a small, yet very well-characterized cohort of participants from the INROAADS study who until now have been clinically classified according to appropriate guidelines by experts in the field and who have been evaluated with a very comprehensive neuropsychological battery as well as a very comprehensive multimodal neuroimaging protocol involving one of the newest radiotracers for astrogliosis. Likewise, given that it is baseline data, there is no missing data on neuropsychological tests. Third, this is the first study coupling the assessment of astrogliosis with relevant growth factors that has provided us the opportunity to address potential mediator effects of astrogliosis in cognition. Further, the assessment of astrogliosis via SMBT-1 PET allowed us to confirm the early role of astrogliosis in some regions such as the precuneus and inferior parietal cortex which are regions of early Aβ accumulation. Moreover, the consistency of ^18^F-SMBT-1 across different brain regions (such as the posterior cingulate, the orbitofrontal cortex and the VLPFC) with worse verbal memory highlight the importance of regional astrogliosis.

However, our results should be considered in the context of several limitations. First, as we have previously mentioned, this is a small sample, with low pathology, which has overall favored the results to the null and constrain classification of participants besides Aβ status. On the other hand, this feature in particular provides us an opportunity to address the very early changes of astrogliosis with this new radiotracer (^18^F-SMBT-1). Second, given that it is an ongoing study, we lack the enrollment of participants with AD, constraining also our findings in the understanding of astrogliosis across the AD spectrum and forcing to study subtle changes that we expect to be more pronounced when considering AD participants. Third, the present study involves a cross-sectional analysis of this single-time measurement of the different biomarkers. Future studies should address the long-term evolution of astrogliosis and of the growth factors evaluated in the aim of confirming and better understanding of the current findings.

## Conclusion

In sum, we found that greater astrogliosis as measured by ^18^F-SMBT-1 PET is associated with worse cognitive performance in nondemented A+ individuals. In addition, we found that IGFR1 may play a neuroprotective role by suppressing the effects of Aβ-related astrogliosis on cognition.

## Supplementary Material

This is a list of supplementary files associated with this preprint. Click to download.

- Suppl.Fig.1.pptx
- Supplemmentarytables12.docx

## Figures and Tables

**Figure 1 A.**
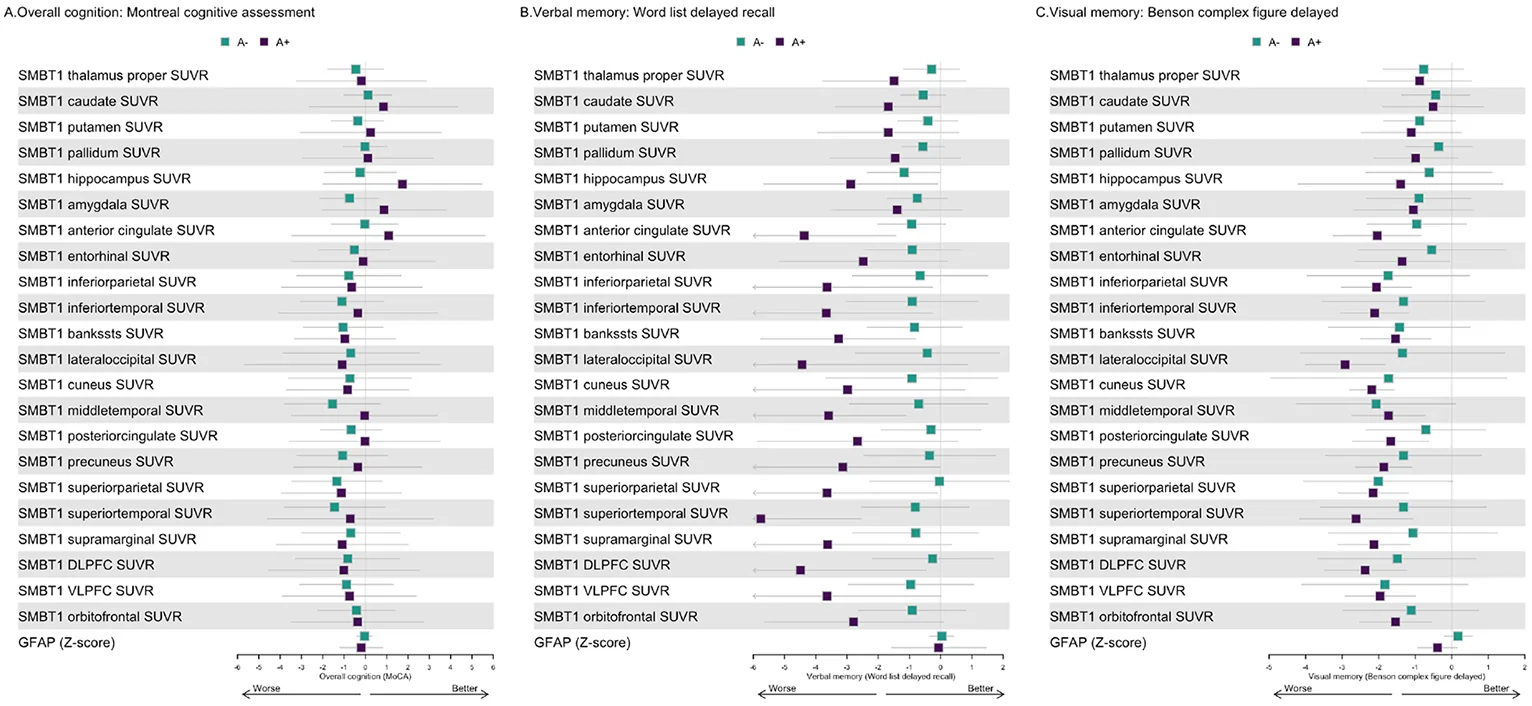
Relationship of ^18^F-SMBT-1 binding and cognitive functions among non-demented A− vs. non-demented A+ participants. Abbreviations: DLPFC: Dorsolateral prefrontal cortex, VLPFC: Ventrolateral prefrontal cortex, BANKSSTS: Banks of the superior temporal sulcus.

**Figure 1 B.**
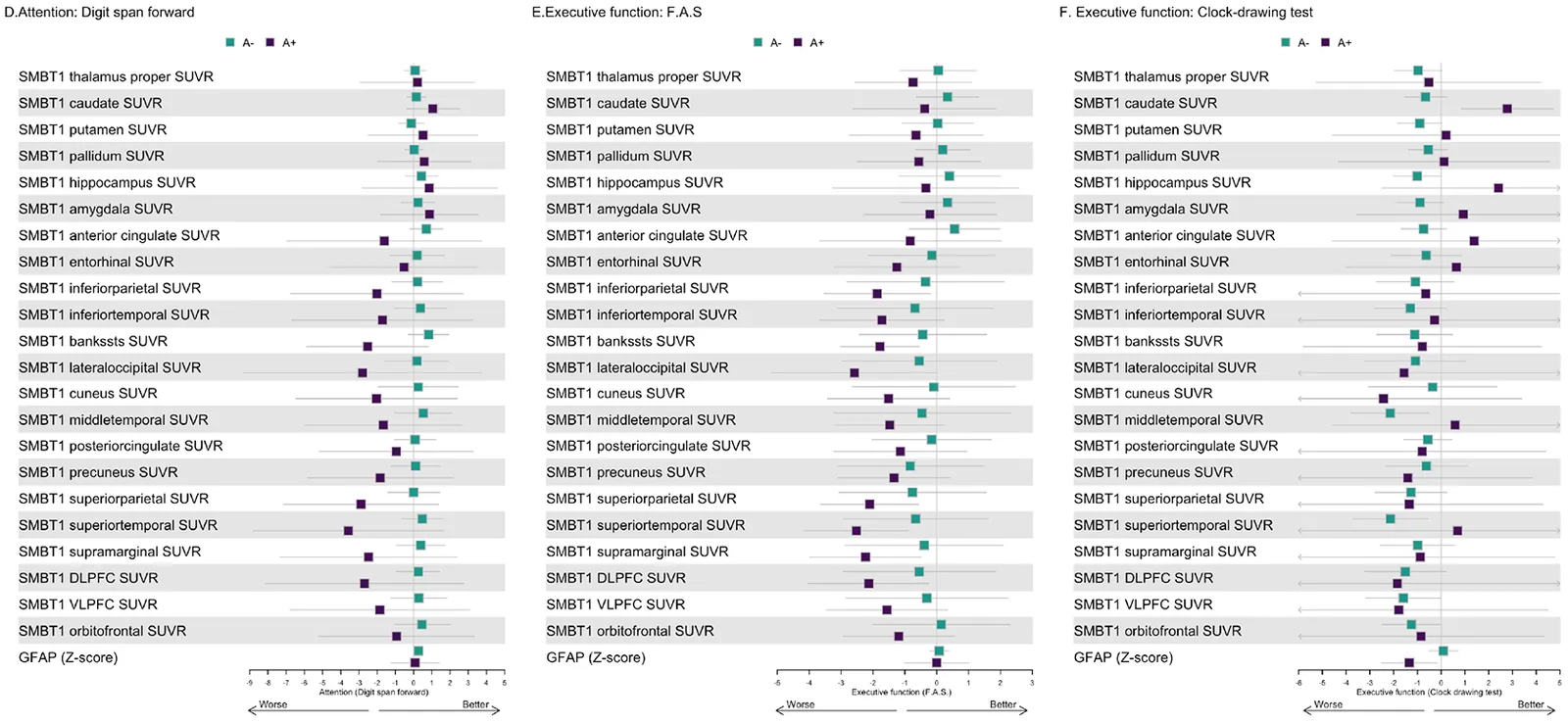
Relationship of ^18^F-SMBT-1 binding and cognitive functions among non-demented A− and non-demented A+ participants. Abbreviations: DLPFC: Dorsolateral prefrontal cortex, VLPFC: Ventrolateral prefrontal cortex, BANKSSTS: Banks of the superior temporal sulcus.

**Table 1:** Characteristics of participants

Age,mean (Std. Dev.)	Whole sample		Non-demented A− (n = 76)		Non-demented A+ (n = 18)		p-val
	68.9	(1.23)	67.9	(0.826)	68.4	(1.629)	0.7979
**Sex**							0.3960
Female	49	(52.1%)	38	(50.0%)	11	(61.1%)	
Male	45	(47.9%)	38	(50.0%)	7	(38.9%)	
**Race**							0.4370
White	84	(89.4%)	67	(88.2%)	17	(94.4%)	
Other	10	(10.6%)	9	(11.8%)	1	(5.6%)	
**Education, mean (Std. Dev.)**	15.95	(0.5)	15.8	(0.28)	16.1	(0.725)	0.6796
**DBP, mean (Std. Dev.)**	75.95	(1.71)	78.4	(1.248)	73.5	(2.165)	0.0785
**SBP, mean (Std. Dev.)**	130.85	(2.46)	133.1	(1.888)	128.6	(3.028)	0.2662
**WML, mean (Std. Dev.)**	2,959.75	(393.9)	3,488	(463.51)	2,341	(324.19)	0.2782
**Aβ burden, mean (Std. Dev.)**	28.1	(4.36)	0.8	(0.783)	55.4	(7.938)	< .0001
**Smoking status**							0.1710
Never smoker	60	(63.8%)	46	(60.5%)	14	(77.8%)	
Former smoker	34	(36.2%)	30	(39.5%)	4	(22.2%)	
**Diabetes**							0.2580
Absent	81	(86.2%)	64	(84.2%)	17	(94.4%)	
Present	13	(13.8%)	12	(15.8%)	1	(5.6%)	
**Hypertension**							0.3430
Absent	46	(48.9%)	39	(51.3%)	7	(38.9%)	
Present	48	(51.1%)	12	(15.8%)	11	(61.1%)	
**Hypercholesterolemia**							0.2240
Absent	38	(40.4%)	33	(43.4%)	5	(27.8%)	
Present	56	(59.6%)	43	(56.6%)	13	(72.2%)	

## Data Availability

The data that support the findings of this study are from Program Grant INROAADS: INflammation ROles in Aging and Alzheimer’s disease Study (P01 AG025204) which available upon request.

## Abbreviations

AD: Alzheimer’s Disease
Aβ: β-Amyloid
PiB-PET: Pittsburgh Compound-B Positron Emission Tomography
CL: Centiloids
GFAP: Glial fibrillary acidic protein
HR: Hazard ratio
95% CI: 95% Confidence Interval
BDNF: Brain-derived neurotrophic factor
VEGF: Vascular endothelial growth factor
IGFR1: Insulin-like growth factor receptor 1
IGFBP7: Insulin like growth factor binding protein 7
MCI: Mild cognitive impairment
CU: Cognitively unimpaired
MAO-B: Monoamoine oxidase B
INROAADS: INflammation ROles in Aging and Alzheimer's disease Study
